# Gathering evidence on preparation for advanced practice in radiation therapy: An international focus group synthesis

**DOI:** 10.1016/j.tipsro.2025.100361

**Published:** 2025-12-01

**Authors:** Yat Tsang, Samantha Skubish, Maria Dimopoulos, Nicole Harnett, Caitlin Gillan

**Affiliations:** a Radiation Medicine Program, Princess Margaret Cancer Centre, Toronto, Canada; b Department of Radiation Oncology, University of Toronto, Toronto, Canada; c Mount Sinai Health System, NY, USA; d Icahn School of Medicine at Mount Sinai, NY, USA; e British Columbia Cancer, Provincial Health Services Authority, Canada; f Division of Radiation Oncology, Department of Surgery, University of British Columbia, Canada

**Keywords:** Advanced Practice Radiation Therapist, Educational Model, Workforce Development

## Abstract

**Purpose:**

Advanced Practice Radiation Therapist (APRT) roles are expanding globally, yet educational preparation approaches vary significantly across jurisdictions. This study synthesized key multidisciplinary interest-holder perspectives to identify essential considerations for APRT preparation.

**Methods and Materials:**

Focus group interviews were conducted via videoconferencing, including practicing APRTs, gatekeepers, educators, and institutional radiation oncology leaders. Semi-structured discussions explored educational content, clinical training, assessment methods, and implementation factors. Data underwent inductive thematic analysis utilizing Braun and Clarke’s six-step approach, followed by concept mapping to organize findings into an integrated thematic structure.

**Results:**

Four focus group sessions involving 33 participants from ten countries across North America, Europe, Asia, and Australia were conducted between October 2024 and January 2025. Five interconnected themes representing key considerations for APRT preparation emerged: (1) content of educational preparation including the four APRT pillars of clinical practice, research, leadership, and education; (2) aligning preparation outcomes with the intended scope of practice, particularly autonomous decision-making and critical thinking; (3) integrating diverse learning processes combining traditional knowledge acquisition with experiential clinical training; (4) key requirements for preparation, including master’s-level education and structured clinical apprenticeship under direct supervision; and (5) critical influence of broader system context on transportability and sustainability across healthcare environments. Participants endorsed master’s-level academic preparation combined with extensive clinical experience, drawing parallels to physician training where apprenticeship builds specialist competencies beyond formal academics.

**Conclusions:**

This internationally derived, multidisciplinary interest-holder synthesis provides foundational guidance for APRT curriculum development, competency standards, and professional credentialing. While these findings offer important direction for standardizing preparation approaches, future research involving broader multidisciplinary interest-holder groups, implementation studies, and outcomes evaluation is necessary to validate and extend these preliminary findings.

## Highlights

• Multidisciplinary synthesis from 33 participants across 10 countries on APRT preparation.
• Four-pillar framework unites clinical practice, research, leadership, education.
• Master’s degree combined with clinical apprenticeship ensures APRT competency.
• Five themes provide foundation for standardized APRT curriculum and credentialing.

## Introduction

Patient-centred care drives modern radiation therapy (RT) practice, emphasizing the need for skilled healthcare practitioners who can deliver complex treatments tailored to individual patient needs [1]. Radiation therapists (RTTs) play a non-replaceable role in cancer patients’ pathway, providing vital education and reassurance to patients during a vulnerable and often frightening time [2].

The Advanced Practice Radiation Therapist (APRT) role represents an advanced level of practice that allows RTTs to work beyond their standard scope through demonstrated expertise in clinical decision-making, leadership, education, and research [3]. Multiple converging factors within RT practice have driven the need for comprehensive APRT educational frameworks. The growing complexity of cancer care and treatment delivery, combined with projected radiation oncology workforce shortages, creates pressure to expand service capacity while maintaining the highest standards of safety and quality care [[4], [5], [6]]. Internationally, APRT implementation has demonstrated measurable benefits for healthcare systems and patient outcomes [[7], [8], [9], [10], [11], [12], [13], [14]]. However, significant educational preparation challenges persist. Understanding the educational and clinical preparation pathways that best equip RTTs for these expanded roles has become essential to meeting evolving healthcare needs and ensuring optimal patient outcomes.

Globally, there is no standardized approach for APRT preparation [14,15]. Regulatory environments, funding mechanisms, scope of practice legislation, and healthcare delivery models vary significantly between jurisdictions, potentially influencing interest-holders' views on optimal educational preparation approaches. Multiple interest-holder groups, including practicing APRTs, gatekeepers, educators, and institutional radiation oncology leaders, may hold different perspectives on optimal preparation pathways.

Given the current lack of consensus on APRT preparation approaches globally and the documented variability in educational backgrounds across jurisdictions, this study forms part of a broader funded research initiative aimed at developing evidence-based, internationally applicable standards for APRT education and preparation [16]. The aim of this qualitative focus group study was to explore multidisciplinary interest-holders' perspectives on the educational preparation pathways required for APRTs, examining what practicing APRTs, gatekeepers, educators, and institutional radiation oncology leaders consider essential for effective role preparation to inform the development of standardized educational approaches for international implementation.

## Methods AND Materials

### Study Design and Participants

This study utilized a focus group approach. Participants were purposively selected to represent one of four main interest-holder groups: APRTs with ≥ 5 years advanced practice experience, gatekeepers (regulatory administrators and professional association representatives), educators in curriculum development, physician mentors and program delivery, and institutional radiation oncology leaders (thought leaders, executive leaders, physician leaders and clinical managers implementing APRT positions). To be eligible, participants needed direct experience with APRT roles or involvement in RTT education, regulation, or leadership. Recruitment was carried out through professional contacts and networks to ensure a range of professional backgrounds and geographic locations (Table 1).

**Table 1:** Focus Group Participant Characteristics and International Representation.

Focus Group	Participants (n)	Coding Convention	Countries Represented	Expertise and Roles Represented
Advanced Practice Radiation Therapist (APRT)	9	AP1-AP9	Canada, United States, United Kingdom, Italy, Australia	Included APRTs with expertise in stereotactic RT, adaptive RT, breast RT and palliative RT.
Gatekeeper	7	G1-G7	Canada, United States, United Kingdom, Australia	Included senior regulatory and credentialing officials, directors of professional standards, and representatives from national and international regulatory and professional committees.
Educator	10 (6 radiation therapists and 4 radiation oncologists)	E1-E10	Canada, United States, United Kingdom, Ireland, Netherlands, Hong Kong, Singapore	Included academic faculty, program directors, and clinical mentors responsible for curriculum development, advanced practice education, and professional training in radiation oncology.
Leader	7 (2 radiation therapists and 5 radiation oncologists)	L1-L7	Canada, United States, United Kingdom, Switzerland	Represented departmental and institutional leaders, including heads of radiation medicine, chairs and vice presidents of oncology, medical directors and RTT professional practice leaders.

### Data Collection

Sessions were led by experienced researchers (SS and YT) and audio-recorded with participant permission. The discussion was guided by a semi-structured interview schedule, developed based on the results of the researchers’ survey study [16] and consultation with subject matter experts in APRT development, workforce planning, and healthcare professional education. Topics included core educational content, learning outcomes, clinical training, assessment methods, and broader factors affecting APRT education (Supplementary Material 1). At the beginning of each session, moderators introduced the study, explained the purpose, set ground rules, and started with open-ended questions. Follow-up prompts were used to encourage deeper discussion and clarify points as needed. Notes were also taken. All participants provided written consent beforehand; their participation was entirely voluntary, and they were free to withdraw at any time. The study was approved by the relevant ethics board (#23–01373). Sessions were audio-recorded with permission and transcribed word-for-word for analysis. Participants were coded by focus group (AP = APRT; G = Gatekeeper; E = Educator; L = Leader) and assigned a participant number within that group (see Table 1 for coding convention).

### Data analysis

Data were analysed using inductive thematic analysis with Braun and Clarke's six-step approach which comprises familiarization, coding, theme development, review, definition, and write-up [17]. The research team (CG, NH, SS and YT) began with repeated reading of transcripts to develop familiarity with the data, progressing from initial readings without annotation to subsequent readings with brief summaries and reflective notes. Meaningful data units were systematically coded by multiple team members, with codes generated inductively from the data. A coding dictionary documenting each code with definitions and exemplars was developed iteratively, with new codes retroactively applied to all data to ensure consistency. Coding discrepancies were resolved through team discussion until consensus was reached.

Codes were organized into candidate themes by identifying semantic and conceptual relationships. Results of each focus group were reviewed individually, then compared across groups to identify shared and unique themes (Supplementary Material 2). Each candidate theme was systematically reviewed for internal coherence, distinctiveness, data support across participants, and relevance to research aims. Themes were split or merged as appropriate. Field notes and debriefs with moderators conducted after each group added contextual understanding during refinement. Before finalizing themes, members not involved in initial coding (MD) reviewed all themes and supporting excerpts. Final themes received concise labels and descriptive statements.

After the initial thematic analysis, a concept mapping exercise was conducted within the research team March 2025 to organize the identified themes into an integrated structure representing key considerations for APRT preparation. Concept mapping is a mixed-methods approach that systematically organises complex data into visual structures revealing relationships between concepts [18,19]. The research team collaboratively reviewed the coded themes generated from the focus groups and organised them into conceptual clusters based on semantic similarity, logical relationships, and functional connections through iterative discussion. This structuring process involved considering how concepts related to each other, which addressed similar aspects of APRT preparation, and how groupings aligned with the study’s research question regarding essential preparation elements. The organised clusters were visually represented in a diagram showing relationships between major themes and their interdependence in developing well-prepared APRTs. Through collaborative interpretation, the research team members discussed what each cluster represented, how clusters related to APRT preparation principles, and implications for educational program design, assigning appropriate labels reflecting the essential meaning of grouped concepts. This team-based approach facilitated expert consensus whilst acknowledging it limited direct interest-holders’ input in the thematic organisation. The finalised thematic structure guided the Results presentation, discussion of implications for curriculum development and credentialing, and identification of future research needs.

## Results

Four focus group sessions involving 33 participants from ten countries across North America, Europe, Asia, and Australia were conducted via secure videoconferencing between October 2024 and January 2025 (Table 1). The Leader focus group session experienced recording failure due to platform error and was documented through detailed co-moderator notes. Each group comprised 7–11 participants, with sessions lasting 60–90 min. The Educator focus group was the largest and most internationally diverse, including 11 members from seven different countries.

**Fig. 1:**
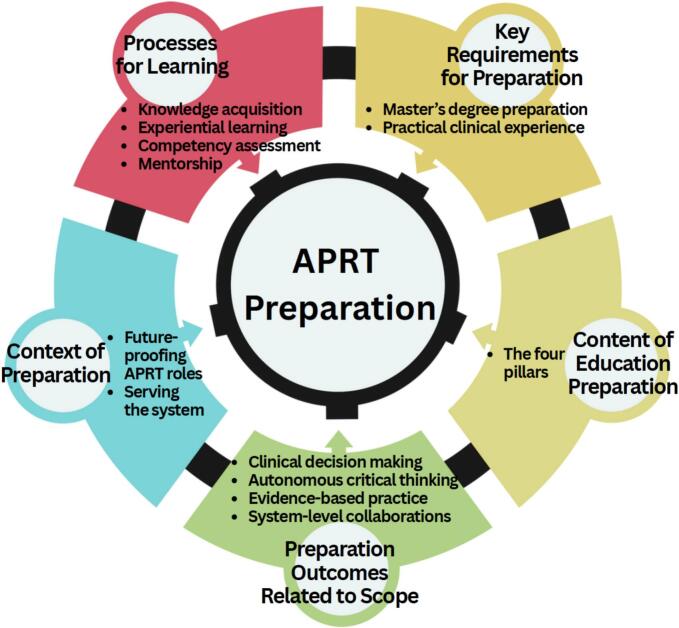
Integrated themes representing interest-holder perspectives on advanced practice radiation therapist (APRT) preparation.

Following thematic coding, a list of 27 coded statements were generated from the focus groups; a five-cluster concept map was developed which guided the inductive analysis during the concept-mapping session. This resulted in five interconnected themes that collectively represent key interest-holder perspectives for APRT educational preparation (Fig. 1). These themes emerged inductively from interest-holder discussions and represent areas of strong consensus across participant groups regarding essential elements of APRT preparation: (1) content of educational preparation including the four APRT pillars, (2) preparation outcomes aligned with intended scope of practice, (3) diverse learning processes integrating multiple pedagogical approaches, (4) key requirements for preparation, and (5) contextual factors influencing sustainability and transportability.

### Content of educational preparation

Reflections on content of educational preparation for APRT practice tended to focus on the four pillars of advanced practice established across multiple national frameworks [3,9,12,13,15]. Participants were not always consistent in the terms used for the specific pillars, but they encompassed the concepts of clinical practice, research, and broader professional skills. Some participants referred explicitly to the existence of four pillars, demonstrating a sense of an emerging standard in common parlance. Those heavily involved with APRT practice, such as APRTs and RTT leaders, had reflections related to how the pillars “go hand in hand to support the practitioner” (E2) with G6’s statement as “I think the approach of the research and development, leadership and management, clinical practice, and education as four pillars − It's very comprehensive in the development of our curriculum.”.

### The Four Pillars

The concepts of clinical, research, and leadership competency were also discussed in detail as being the critical elements of what should be addressed in preparing for APRT practice. In the clinical domain, there was a sense of the value of appreciating “the full patient pathway” (L2), for the oncology patient, but also broader “general medical knowledge” (AP5) extending beyond the oncology domain. As further noted by AP5, “And I think it's valuable to have the radiation oncologist kind of teach us the decision-making, you know, the treatment factors, tumour factors, patient factors. Those are, truly important.”. There was also discussion about the scope of clinical learning for APRTs. While participants supported a unified educational foundation, they consistently stressed the importance of allowing practitioners to focus and develop expertise within their specific area of clinical practice. In addition, competencies related to research, such as critical appraisal of the literature (AP4), leveraging of evidence-based practice (AP4, L6), and conducting research projects (L6), were considered essential components of the overall curriculum. These research skills were valued not only as distinct areas of learning, but also as integral to clinical and leadership competency in which APRTs would establish the “innate ability to sum some knowledge of research and education, so that they are of value to their colleagues” (E4).

Reflections on leadership were heavily focused on distinguishing between being a leader and holding a management position, especially within the Leader focus group. Having the ability to lead was believed to be an important element of APRT preparation and related strongly to mastery of other pillars. This was encapsulated as being a good team member, an independent thinker, and a collaborator (L1), as well as a change champion who could appreciate how RT integrates with other modalities and services to the benefit of the patient (L2). With respect to practical skills in this domain, AP5 reflected that things like “project management…is not part of my program, my previous training. And I felt afterwards I had to learn it [as part of a master’s program related to APRT].”.

### Preparation outcomes related to scope

While there may have been overlap in how related concepts were discussed, there was distinction between the content and outcomes of educational preparation. Four main outcome areas were distilled, which exhibited some inter-reliance and clear alignment with the content ‘pillars’ – competence in clinical decision-making, autonomous critical thinking, evidence-based practice, and system level collaboration.

### Clinical Decision-Making

Clinical decision-making was the most distinctly clinically focused outcome and was discussed in terms of an elevated ability to pull together a broader and deeper scope of information (than would be accessible to RTTs) to guide patient management. AP4 and AP5 noted it to be one of the most valuable outcomes of their masters’ programs when training to become APRTs, specifically that they became able to manage the “rationalization and justification for decision-making where you call upon the evidence“ (AP4).

### Evidence-Based Practice

While it could be considered an extension of clinical decision-making, reflections on evidence-based practice as an outcome of APRT preparation went beyond clinical management of individual patients, extending into the ability to leverage and create evidence to the betterment of the system (AP3, AP5). Specifically, AP3 referenced the APRT’s ability to recognize their role in “service improvements, [and] how to do them”.

### Autonomous Critical Thinking

An aligned outcome concept was autonomous critical thinking; holding unique accountability and working independently with advanced knowledge and understanding of the practice context. This was often referenced in terms of having a better appreciation of the self – self-confidence (E4), self-awareness (E2) and self-directedness (E2). As noted by L7, this outcome pertained to being able to think strategically including the full cancer management course of the patient. A distinguishing characteristic of effective APRT roles was seen to be that they “not be [built] around people or certain technologies and [be] more about the sort of autonomous practice and the decision-making” (G4). This was also felt to require an elevated appreciation for one’s accountability to the patient given this level of autonomy in practice, cultivated through educational preparation (AP4).

### System Level Collaboration and Integration

An outcome aligned with leadership competence was the concept of system level collaboration and integration. E7 framed this simply as working in “partnership” with the radiation oncologists and other team members. Two participants in the APRT group illustrated this more fulsomely as follows:

The confidence to reach out to lots of different kind of departments and areas of people…get my foot in the door and go, you know, sit in the clinics or talk to a cardiothoracic surgeon (AP3).

Understanding outside of radiation therapy lens, in terms of palliative and hospice care…has given me a different perspective and a different viewpoint on looking at patients, being able to navigate palliative patients, speaking a language that is outside of radiation therapy (AP1).

### Processes for learning

Participants often referred to the types of activities that might be required of an individual to build and evidence competency as an APRT, separate from the specific areas of knowledge or skill that needed to be acquired. These activities could be categorized as knowledge acquisition, experiential learning, competency assessment and mentorship. None of these learning activities were mutually exclusive, though all were mentioned as being necessary to the preparatory journey for an APRT, supporting them in acquiring the full complement of advanced knowledge, skills, and judgement.

### Knowledge Acquisition

Knowledge acquisition was highlighted, specifically as the work done by way of a traditional, lecture-based approach (G5, G6). E9 specifically mentioned the concept of “learning to learn”, especially given that many pursuing APRT had been working as RTTs for several years and may have been out of practice in knowledge acquisition in a formal teaching setting.

### Experiential Learning

Learning in the clinical environment was deemed a critical complement to foundational didactic learning, serving to consolidate the knowledge base through case-based learning, following patients, applying knowledge, and observing clinicians in practice (AP3, E1). This was often noted by those in the APRT and the Educator focus groups. E2 stated “So it's not just a ‘you're competent’ one day, and yes, you've ticked the box. It's a build up of numerous pieces of evidence, so it might be a case-based discussion, it might be being observed, it might be a multi source feedback. So there would be numerous different things that are used. So what we're doing is thinking about how the practitioner develops capability, not just competency.”. E4 acknowledged the distinct value of experiential learning in providing the real-time opportunity to assess critical thinking and clinical decision-making skills as per statement “The important thing in assessing clinical ability is direct observation. Now I know that students in general find that a very challenging situation, but I think that there has to be at least some element of direct observation. I think you can't really assess it purely in a written format.”.

### Competency Assessment

Relatedly, another element was how competence should be demonstrated through practical competency assessment, including structured observation and feedback (L3, E4, E9), the use of portfolios (G5, AP2), and ensuring alignment with APRT competency profile (G5, AP1, E9). AP2 reflected that having a formalized competency profile allowed individual learners to self-assess their progress as they worked to integrate themselves in a clinical role through experiential learning, especially when no formalized assessment structure existed.

### Mentorship

Parallel to explicit curriculum and assessment, mentorship was also felt by many, especially those in the APRT group, to be essential. This was framed primarily as direct mentorship by a radiation oncologist, related to teaching, observing, and modelling elements of the APRT role in the clinical environment (AP4, AP6, E7, E8), but also mentorship by other APRTs (AP4, E8).

The concept of an APRT community of practice, established in Canada but in place internationally at the time of this study, was acknowledged as an element of mentorship that would contribute to ongoing learning supported by “Communities of practice and support systems are really important…I know that we’re talking about mentorship and how we can build on those relationships, and I think that would be a very, very valuable part for someone getting into the role.“ (AP4).

An underlying aspect of learning and competency, frequently referenced across all focus groups, was the process by which RTTs develop and refine their individual scope of practice as APRTs. This involves applying the established competency framework to their specific clinical contexts—guided primarily by experiential learning, but also supported by knowledge acquisition and mentorship. Both self-directed (AP2, AP3, AP4) and collaborative (G2, E3, E5) approaches were described as important for clarifying the precise boundaries of the role, and for identifying the skills and knowledge to be acquired and assessed. These reflections emphasized that adaptation occurs within the broader professional scope, rather than fundamentally redefining the APRT role for each practitioner, this is supported by “[We must] be confident in our limitations, know where we are fitting into the pathway and picture, and also to make the people around us, both the patients and also the clinicians, whoever it might be, they know where we fit.“ (AP3).

### Key requirements for preparation

Significant time in all focus groups involved consideration of what were viewed to be the two foundational elements of educational preparation for APRTs; completion of a graduate program (specifically, a master's degree) and the need for practical clinical experience. There was a lack of consensus on whether these two components needed to be linked into a single entity, or whether they could be accessed separately.

### Master’s Degree Preparation

Most participants deemed master’s level education necessary, with justification relating to many earlier themes; acquiring a knowledge base according to APRT pillars, building foundations for critical thinking and clinical-decision making, and honing research skills and an appreciation for evidence-based practice. Many in the Educator focus group referred specifically to building knowledge and learning to apply it in an academic setting, in that achieving a “master's and above [can serve to] ascertain that they do have that level of critical thinking and ability [with] the academics behind it” (E7). Similarly, it could “prepare them for the clinical piece. You know, I think you need to build up a foundation of knowledge, and that might be through, you know, formal teaching, didactic teaching, and so forth” (E5). AP5 believed clinical decision-making to be the most valuable outcome of their master’s experience that related to their future as an APRT.

The role of a master’s program in building research skills was noted more by those in the Leader focus group (L3, L6, L7), where it was articulated that a dedicated lens to evidence-based practice would not have been a focus in an entry-to-practice baccalaureate program. It was in the Educator focus group, however, that this lens was framed explicitly as building “research literacy” (E2, E6, E9). A master’s program was believed to socialize APRT learners to self-directedness in learning, important in ultimately building such tailored competency, as supported by “Mentally I still had this expectation, we’re all going to sit there and be taught stuff, and it was very much an eye-opening experience to be like, I’m going to teach me this stuff. They’re providing the framework for I guess, like sticking the bricks on that learning.“(AP4).

It was also noted that a graduate degree allowed for an externally mandated standard to be implemented. This was only mentioned in the Gatekeeper and Leader focus groups, and it was a topic of lengthy discussion. The consensus was that without a mandated minimum, “standards tend to get eroded over time” (L2) and that given that a profession has an obligation to set a clear bar, a master’s degree is a reasonable expectation (G4).

Despite this agreement regarding a master's degree as a standard, there were concerns about the ability to mandate it. This was attributed to smaller volumes of APRTs and limited number of focused programs and related accreditation resources. Those in the Gatekeeper focus group suggested it would not be within their purview to weigh into education requirements for eligibility for certain classes of practice (G1, G6). There were concerns about perceived feasibility of a tailored educational program for APRTs (G3, G6). It was noted that any graduate degree would ensure “some generic skills”, such as “critical thinking” (L3). Others felt a degree should be framed at the least within radiation oncology (G2, G4), or in an aligned area, such as healthcare quality or leadership, and could then be applied to APRT later (L7).

### Practical Clinical Experience

Many participants across all focus groups considered practical experience in the intended clinical environment to be as important as, or even more important than, obtaining a graduate degree. L5 suggested that the two could happen in tandem, while E5 noted explicitly the value of leveraging such an experience later to benefit from consolidation of knowledge in the context of their role.

L3 drew a comparison to physician training, highlighting that the essential component for developing “specialist skills” is the clinical apprenticeship under direct supervision, rather than the completion of a graduate (i.e., master’s-level) degree. The point was made that, while some formal academic background is required, most physicians undertake undergraduate-entry medical degrees rather than graduate studies, and their clinical competence is built primarily through extensive hands-on training. In this context, participants suggested that, just as for radiation oncologists, clinical exposure is indispensable for APRTs: “for those who wish to have some clinical skills, there is no substitute for actually putting them in the clinic” (E4).

Immersion in the clinical practice environment was also a rich mechanism to support role development (A5). The concept of a portfolio was mentioned to measure clinical integration in a structured way, in that to be deemed competent, “They have to once again show that they've been working as an advanced practitioner in the field for that period of time in that particular area and they need to be able to show supporting evidence [at] an organization supporting that particular role.“ (G5).

### Context of preparation

APRT educational preparation was suggested to be adaptable and sustainable in rapidly evolving healthcare environments. Participants agreed that frameworks should equip APRTs with broad, foundational competencies that support autonomous practice and flexible role adaptation, ensuring relevance and transferability across institutions and countries.

### Future-proofing APRT Roles

The importance of designing educational programs that enable APRTs to remain current and responsive to ongoing changes in the field was emphasized. As highlighted by L7, “making sure your role is always meeting the needs—staying current and adapting role.”.

The rapid pace of technological innovation was frequently cited as a driver for programs to focus on adaptability and critical thinking, rather than training for specific tools or protocols. G5 explained, “One of the things is future-proofing where we go, irrespective of what it looks like….…So whilst we’re all operating now, developing all these programs, we’re actually looking at the cohort that’s not immediate. It’ll be the future point of time.... I wish I had a crystal ball, but also looking at what technology is going to change, how it’s going to affect what we do as a professional, and where our scopes could expand to.”.

Also from the Leader focus group, G3 emphasized the need for broad, flexible education that encourages innovation: “Practice is changing rapidly, and I think there’s a lot of opportunities for AP roles beyond the areas that we’ve already developed so far. So I think the education must be quite broad and give the people that are taking the program the opportunity to be influenced in as many ways as possible by pushing the envelope on practice.”.

### Serving the System

In addition to future-proofing the profession, participants in the Gatekeeper focus group emphasized that APRT educational preparation should address broader system needs. They agreed that programs should focus on “encouraging roles to not be around people or certain technologies and more about the sort of autonomous practice and the decision-making” (G4).

Thought to be critical to sustainability was the concept of transportability facilitating APRT movement across various institutions and even internationally. As G2 explained, “If we can have something in place that’s more general, I think other cancer centres might be able to use that to develop their needs, and then we can see something that’s transportable. But waiting for somebody to have a need is extremely difficult in terms of trying to grow an APRT.” G5 reinforced this point: “We need to start looking at how whatever package it is that that practitioner takes is transportable to wherever they go and potentially transportable [across] countries as well.”.

## Discussion

This multidisciplinary focus group study identified five interconnected themes representing interest-holder perspectives on essential considerations for APRT preparation: the need for the content of educational preparation including the four APRT pillars, the importance of aligning preparation outcomes with the intended scope of practice, the significance of integrating diverse learning processes, considerations around the key requirements for preparation, and the critical influence of the boarder system context.

Participants consistently endorsed the four-pillar principles (clinical practice, research, education, and leadership) as the core to APRT development and professional credibility. This aligned with the established frameworks in the international contexts, where dynamic integration of these pillars enables practitioners to adapt to shifting technologies and clinical paradigms, ensuring professional acceptance across disciplines and sustained career growth [3,8,9,14,15].

One of the main findings from this study was the importance of ensuring that preparation outcomes are closely aligned with the intended scope of APRT practice, particularly in relation to autonomous decision-making, critical thinking, and an evidence-based approach. These attributes are increasingly counted as prerequisites for APRTs facing complex, rapidly evolving treatment modalities and multidisciplinary care management. As evidenced by examples in Australia, Canada, the United Kingdom and the United States, APRTs with advanced decision-making and analytical skills have demonstrated measurable improvements in patient outcomes, safety, and collaborative team functioning [3,[7], [8], [9], [10], [11]]. The ability to perform autonomous clinical assessment, formulate treatment recommendations, and implement evidence-based interventions represents a fundamental shift from traditional task-oriented roles to comprehensive patient-centred practice [3,14,15].

The appreciated need for diversity in learning approaches, including knowledge acquisition, experiential learning, and mentorship highlighted the complexity of advanced practice competencies that cannot be adequately developed through traditional classroom instruction alone. Roumeliotis et al suggested that combining structured academic preparation with mentored clinical experiences produces practitioners who are better prepared for complex decision-making and autonomous practice [20]. Portfolio assessments could be utilized as a powerful tool for validating complex skill acquisition, supporting reflective practice, and enabling individualized learning [21].

The finding that might merits most attention is the role of master’s level education. This work served to reinforce expert consensus on the need for graduate education, already evidenced in practice frameworks in many jurisdictions. However, consistent with the findings of the systematic review by Oliveira et al, it fell short of articulating a clear view on the appropriate scope of graduate study [15]. Future work considering the requirements of a master’s degree could also draw from the recognition (as per the final theme elucidated in this work), that the nature of feasible and responsible APRT educational preparation depends heavily on system-level factors including regulatory frameworks, funding mechanisms, organizational support, and healthcare delivery models. These contextual factors significantly influence educational preparation requirements, scope of practice development, and role sustainability. The APRT roles can only function in their maximum potentials in environments where mentorship, clear career pathways, and responsive support structures are prioritized, and where educational innovation is met with progressive regulatory and credentialing strategies [14,15,22].

Strengths of this study include the international scope spanning 10 countries and the inclusion of diverse interest-holder perspectives across multiple professional roles. Notably, 9 radiation oncologists participated across the Educator and Leader focus groups, providing multidisciplinary clinical perspective on the knowledge, skills, and competencies required for APRT roles. This physician input is particularly valuable given the emphasis participants placed on radiation oncologist mentorship in clinical training and the parallels drawn to physician apprenticeship models for specialist skill development. The inclusion of regulatory professionals alongside practicing APRTs and educators enabled examination of perspectives from those who set standards, deliver education, practice in advanced roles, and implement positions within healthcare organizations. The focus on experienced and engaged individuals from primarily English-speaking jurisdictions (without representation from low- and middle-income countries) may limit the generalizability of findings to diverse cultural and healthcare contexts. However, the alignment of results with established international frameworks and published literature supports the transferability of core principles identified. The concept mapping approach, while valuable for theme development, was conducted within the research team rather than with participants, potentially limiting interest-holders’ input in the thematic organisation [18,19].

## Conclusion

This study gathered multidisciplinary interest-holder perspectives on effective APRT preparation and identified consensus around a structured four-pillar foundation that prioritizes the development of critical thinking and autonomous clinical decision-making. Educational elements such as knowledge acquisition, experiential learning, and mentorship were suggested to be achievable through a combination of graduate education and a formalized practical clinical experience. Successful APRT educational program development requires careful attention to contextual variables that influence implementation and sustainability across different healthcare systems. Future research should focus on validating and extending these insights through longitudinal outcome studies, comparative model evaluation, and implementation research across diverse healthcare contexts. Ultimately, the effectiveness of APRT education internationally should be measured by its impact on patient outcomes, healthcare team performance, and sustained professional advancement within radiation oncology practice.

Disclosure:

Research reported in this study is supported by the American Society of Radiologic Technologists Foundation through the International Collaborative Research Grant. 

## Declaration of competing interest

The authors declare that they have no known competing financial interests or personal relationships that could have appeared to influence the work reported in this paper.

## Data Availability

Data will be made available on request. The data that support the findings of this study (e.g., deidentified focus group transcripts and coding frameworks) are available from the corresponding author upon reasonable request. Data are not publicly available due to privacy and ethical restrictions related to participant confidentiality.
