# Supplementary material for: Gathering evidence on preparation for advanced practice in radiation therapy: An international focus group synthesis
Source: Tech Innov Patient Support Radiat Oncol. 2025 Dec 1;36:100361. doi: 10.1016/j.tipsro.2025.100361 (PMC12722987; doi:10.1016/j.tipsro.2025.100361)
Supplement: Supplementary Data 1 [file mmc1.docx]

# Supplementary Material 1 - Semi-Structured Focus Group Interview Guide

## Study Information

**Study Title:** Conducting an international SWOT analysis to identify the appropriate framework for APRT preparation in the US and Canada

**Principal Investigator:** Maria Dimopoulos, PhD, MBA, RT(T)

**Ethical Approval:** Study #23-01373 (Mount Sinai Health System Institutional Review Board)

## Session Overview

The study explored multidisciplinary interest-holder perspectives on educational preparation pathways for Advanced Practice Radiation Therapists across international healthcare contexts. Four separate focus groups were conducted between October 2024 and January 2025, each representing a distinct stakeholder perspective:

- APRT: Practicing Advanced Practice Radiation Therapists with 5+ years experience
- Gatekeeper: Regulatory administrators, professional association representatives, credentialing officers
- Educator: Radiation therapist educators, curriculum developers, academic program leaders, physician mentors
- Leader: Institutional radiation oncology leaders, radiation oncologists, executive leaders, clinical managers

## Focus Group Conduct Protocol

### Opening Procedures

Each session began with facilitator introductions and an overview of the study purpose. Participants were reminded of confidentiality protections and that their participation was voluntary. Ground rules emphasized respect for diverse perspectives, with one person speaking at a time to facilitate accurate recording. After confirming written informed consent, participants were informed that audio recording would capture discussion for later transcription, with all identifying information removed during analysis.

## Semi-Structured Interview Guide

The interview guide was developed iteratively based on results from the research team's preceding survey study and consultation with subject matter experts in APRT development, workforce planning, and healthcare professional education. The guide explored five major domains while remaining flexible to allow participants to raise additional topics of importance.

### Domain 1: Content of Educational Preparation for APRTs

**Opening Question:** "When you think about preparing someone to work as an Advanced Practice Radiation Therapist, what are the essential knowledge and skill areas they need to develop?"

Follow-up probes explored clinical knowledge requirements (breadth versus specialized depth), the importance and types of research skills needed, the role of leadership development in APRT preparation, how different knowledge domains relate to each other, and whether content needs differ by clinical specialization (e.g., stereotactic radiosurgery, adaptive therapy, palliative care).

### Domain 2: Learning Outcomes and Scope of APRT Practice

**Opening Question:** "What do you want an APRT to be able to do after completing their preparation?"

Probes addressed clinical decision-making and autonomy (what decisions should APRTs make independently), how APRTs should differ from traditional radiation therapists in their approach to patient care, the role of evidence-based practice in APRT work, how APRTs interact with the broader multidisciplinary team, and what specific competencies differentiate autonomous APRT practice.

### Domain 3: Learning Processes and Pedagogical Approaches

**Opening Question:** "How do APRTs best learn and develop the knowledge and skills needed for their role?"

Discussion explored the role of formal academic instruction versus clinical experiential learning, whether structured clinical apprenticeship or mentorship should be included, who should mentor APRTs (radiation oncologists, other APRTs, or others), how competency should be assessed and demonstrated, and what assessment methods would be appropriate (e.g., written exams, practical assessments, portfolios, case-based discussions, direct observation).

### Domain 4: Formal Educational Requirements and Pathways

**Opening Question:** "What educational qualifications or requirements should APRTs have?"

Probes examined whether graduate education (e.g., master's degree) should be required, what field or discipline the degree should be in, whether graduate education and clinical experience should happen simultaneously or sequentially, how much clinical experience APRTs should have before and during preparation, and what barriers exist to implementing standardized educational requirements across different jurisdictions.

### Domain 5: System-Level Factors, Sustainability, and Context

**Opening Question:** "What factors in the healthcare system or broader environment influence how APRT education is designed and implemented in your setting?"

Discussion addressed how regulatory or credentialing requirements affect APRT preparation, the role of funding mechanisms in educational accessibility, how important it is that APRT roles and preparation be consistent across different institutions or countries, what should be considered to ensure APRT roles remain relevant as technology and practice evolve, and what system-level support is needed for APRT sustainability and career progression.

### Closing Questions

Sessions concluded by asking participants if there was anything else about APRT educational preparation that had not been discussed, and what their top priorities would be for standardizing APRT preparation internationally.

## Facilitation Approach

Sessions were led by experienced researchers (SS and YT) using an open-ended questioning approach that allowed participants to express perspectives freely. Follow-up prompts encouraged deeper discussion and clarified points as needed. Participants were invited to build on colleagues' comments or respectfully disagree. Discussions were audio-recorded and supplemented with field notes taken by a co-facilitator. Sessions lasted 60-90 minutes.

## Adaptation by Focus Group

While the core interview guide remained consistent across all focus groups, minor language adaptations ensured relevance to each stakeholder group. For APRTs, questions referenced their personal experience becoming an APRT and challenges they faced. For Gatekeepers, emphasis was placed on standards, regulation, credentialing, and accountability. For Educators, questions focused on curriculum design, learning outcomes, assessment methods, and accreditation. For Leaders, emphasis was placed on institutional context, workforce planning, sustainability, and system-level integration.

## Post-Session Procedures

### Immediate Debrief

Facilitators conducted a brief debrief immediately after each focus group to verify completeness of field notes, clarify any ambiguous participant statements, and add contextual observations about group dynamics.

### Transcription and Verification

Audio recordings were transcribed word-for-word by a professional transcription service. Transcripts were reviewed by facilitators for accuracy and completeness. Participant identifiers were anonymized using the coding convention (AP1-9, G1-7, E1-10, L1-7). The Leader focus group experienced platform recording failure due to technical error and was documented through detailed co-moderator notes taken during the session, supplemented by post-session facilitator debrief.

### Data Security

All recordings and transcripts were stored securely with access limited to the research team. Participant identities were protected through use of the coding convention, and no identifying information was included in transcripts or analytical materials.
