# Supplementary material for: Gathering evidence on preparation for advanced practice in radiation therapy: An international focus group synthesis
Source: Tech Innov Patient Support Radiat Oncol. 2025 Dec 1;36:100361. doi: 10.1016/j.tipsro.2025.100361 (PMC12722987; doi:10.1016/j.tipsro.2025.100361)
Supplement: Supplementary Data 2 [file mmc2.docx]

# Supplementary Material 2 - Detailed Thematic Analysis Methodology and Development of Themes

## *Overview of Analytical Process*

Following inductive thematic analysis using Braun and Clarke's six-step approach applied to four focus group transcripts, data were systematically coded and organized into themes. This document provides detailed methodological information about the thematic analysis process, including how 27 individual codes were developed and subsequently organized through concept mapping into five interconnected themes representing key considerations for APRT educational preparation.

## *Braun and Clarke Six-Step Thematic Analysis Process*

### Step 1: Familiarization with the Data

The research team (CG, NH, SS, YT) began with repeated reading of transcripts to develop familiarity with the data. Initial readings were conducted without annotation to gain holistic understanding, progressing to subsequent readings with brief summaries and reflective notes on interesting patterns, contradictions, or unexpected findings. Field notes from facilitators and debriefs conducted after each focus group were reviewed to add contextual understanding. The research team met to discuss their initial impressions and observations across all four datasets. This immersive familiarization process was essential given the international, multisite nature of the study and the importance of understanding context-specific language and perspectives from participants across ten countries.

### Step 2: Systematic Coding of Data

Meaningful data units were systematically coded by multiple team members (CG, NH, SS, YT). Coding was inductive, meaning codes were generated directly from the data rather than imposed from existing frameworks. All team members coded independently from at least one complete focus group transcript to develop individual coding frameworks. Codes were generated at both semantic (explicit meaning) and latent (underlying concepts) levels. Initial coding focused on capturing discrete ideas, statements, or concepts that appeared relevant to APRT educational preparation.

### Step 3: Development of Coding Dictionary and Iterative Application

After initial coding, the research team collaboratively developed a coding dictionary documenting each identified code with concise code names, clear definitions, exemplar quotes from the data, and exclusion criteria. The developed codebook was applied to all transcripts systematically. When new codes emerged during this process, they were added to the codebook with full documentation, and previously coded data were reviewed and re-coded with new codes as appropriate to ensure consistency. This iterative process ensured that the entire dataset was coded according to the final, comprehensive codebook. A total of 27 coded concepts were identified and systematically applied across all focus group data.

### Step 4: Organization of Codes into Candidate Themes

The research team reviewed all identified codes and began grouping them based on semantic and conceptual relationships. Semantic grouping clustered codes that shared explicit, surface-level meanings together. Conceptual grouping organized codes that shared underlying concepts or represented different manifestations of similar ideas. Candidate themes were developed by identifying the broader pattern or concept that unified each group of codes. Initial candidate themes addressed educational content and core pillars of APRT development, preparation outcomes and scope of autonomous practice, types of learning processes and pedagogical approaches, educational requirements and pathways, and system-level contextual factors.

### Step 5: Review and Refinement of Themes

Each candidate theme was evaluated for internal coherence (whether codes cohered meaningfully with a clear unifying concept), distinctiveness (whether the theme was clearly distinct from other themes and represented a unique pattern), data support (whether the theme was supported by data across multiple participants and ideally across multiple interest-holder groups), and relevance to research aims (whether the theme addressed the study's research question about APRT educational preparation). Results of each focus group were reviewed individually to identify themes specific to that group, then compared across groups to identify shared themes, unique themes, and points of divergence or differing emphasis among groups. A team member not involved in initial coding (MD) reviewed all finalized themes and their supporting excerpts to ensure themes accurately represented the data, quotes appropriately exemplified the theme, and no important data were omitted or misrepresented. Any discrepancies or concerns raised were discussed and resolved by the full research team.

### Step 6: Final Definition and Write-Up of Themes

Each final theme received a concise, descriptive label reflecting its core concept. A detailed definition was written for each theme explaining what the theme represents, why it matters to APRT educational preparation, and how it relates to the research question and study aims. Rich illustration from the data supported each theme in the final write-up.

## *Bias Mitigation Strategies*

Several strategies were employed to minimize researcher bias and ensure analytical rigour:

### Team-Based Coding

Multiple team members (CG, NH, SS, YT) independently coded data before collaborative discussion. This approach reduced reliance on any single researcher's interpretation, allowed diverse perspectives to emerge, and ensured no single researcher's biases dominated the analysis.

### Inter-Rater Consensus and Conflict Resolution

When individual coding varied, the team discussed discrepancies using the codebook definitions. Consensus was reached through collaborative discussion rather than majority vote. Differences were documented to understand whether they reflected ambiguity in the codebook requiring refinement, genuine ambiguity in the data, or different but equally valid interpretations. Resolution prioritized data-driven evidence rather than researcher opinion.

### Bracketing and Reflexivity

The research team acknowledged their own backgrounds and potential biases. Several team members had experience with APRT roles in their own countries, which was valuable for understanding context but could introduce bias toward certain perspectives. Regular discussions were held to identify and discuss potential biases. Field notes included facilitator reflections on their own reactions during sessions.

### Independent Review

A team member (MD) not involved in initial coding provided independent verification. This fresh perspective helped identify potential bias in theme interpretation and assessed whether themes were over-interpreted or under-supported by data.

### Audit Trail

Complete documentation was maintained of initial coding frameworks developed by each team member, codebook evolution (original codes, new codes added, codes merged), decisions about theme refinement (splitting, merging, renaming), and conflict resolution discussions. This audit trail provides transparency and allows verification of analytical decisions.

### Attention to Negative Cases

During theme development, the team actively looked for data that contradicted or partially contradicted emerging themes, perspectives that diverged from consensus, and alternative explanations for observed patterns. These were examined carefully to ensure themes accurately represented the full range of participant perspectives. While most participants endorsed master's-level education, concerns about feasibility and accessibility (particularly in smaller jurisdictions) were given equal analytical weight.

## *Summary of 27 Individual Codes Identified During Thematic Analysis*

Through systematic inductive coding, 27 distinct codes were identified across all focus group data. These codes represent discrete concepts, patterns, and ideas related to APRT educational preparation. Table 1 provides a complete list of all 27 codes organized by their primary thematic cluster.

Table 1: Complete List of 27 Codes Identified Through Inductive Thematic Analysis

| Code # | Code Name | Primary Thematic Cluster |
| --- | --- | --- |
| 1 | Self-development, autonomy, self-actualization | Preparation Outcomes |
| 2 | Role definition, distinction, development | Preparation Outcomes |
| 3 | Leadership & research as key components | Content of Education |
| 4 | Master's level education as the standard | Key Requirements |
| 5 | Systematic methods of assessment beyond competency | Learning Processes |
| 6 | Content of education | Content of Education |
| 7 | Pillars of APRT (clinical, research, leadership, education) | Content of Education |
| 8 | Autonomous critical thinking | Preparation Outcomes |
| 9 | Level of education | Key Requirements |
| 10 | Work experience beyond entry-to-practice | Key Requirements |
| 11 | Demonstration of learning | Learning Processes |
| 12 | Common standard | Key Requirements |
| 13 | Need to evidence AP through experiential learning & assessment | Learning Processes |
| 14 | Ability to serve the system | Contextual Factors |
| 15 | Future-proofing: adaptability to changing practice | Contextual Factors |
| 16 | Transportability: general vs specialization | Contextual Factors |
| 17 | Deepening the knowledge base beyond radiation therapist training | Content of Education |
| 18 | Medical foundations for APRTs | Content of Education |
| 19 | Specialized oncology knowledge | Content of Education |
| 20 | Strengthening clinical reasoning and decision-making skills | Preparation Outcomes |
| 21 | Improving confidence in decision-making and managing uncertainty | Preparation Outcomes |
| 22 | Experiential learning: structured immersion in the clinical environment | Learning Processes |
| 23 | Structured clinical immersion | Learning Processes |
| 24 | Leading evidence-based care through research and quality improvement | Learning Processes |
| 25 | Communication across disciplines through collaborative leadership | Learning Processes |
| 26 | Promoting reflective practice and ongoing growth | Preparation Outcomes |
| 27 | Adaptability and accessibility of APRT education program (including expert mentorship) | Contextual Factors |

These 27 codes were developed inductively from the data and systematically applied across all four focus group transcripts. Each code was defined in the coding dictionary with clear definitions, exemplar quotes, and exclusion criteria to ensure consistent application by all team members.

## Concept Mapping Process

After the initial thematic analysis, a concept mapping exercise was conducted within the research team in March 2025 to organize the 27 identified codes into an integrated structure. Concept mapping is a mixed-methods approach that systematically organizes complex data into visual structures revealing relationships between concepts.

### Preparation and Structuring

The research team reviewed all 27 codes and their supporting data. A collaborative session generated discussion about how key concepts and ideas related to APRT preparation connected to each other. The 27 codes were organized into conceptual clusters based on semantic similarity (concepts with similar meanings grouped together), logical relationships (concepts that logically connect or build on each other), and functional connections (concepts related by their role in APRT preparation). Iterative discussion allowed the team to consider how concepts related to each other and which addressed similar aspects of APRT preparation.

### Representation and Interpretation

The organized clusters were represented showing major conceptual clusters (five themes), relationships between clusters, and sub-concepts within each cluster supporting the main theme. The research team discussed what each cluster represented conceptually, how clusters related to broader APRT preparation principles, implications of each cluster for educational program design, and whether clusters addressed content, process, requirements, or context.

### Labeling and Utilization

Appropriate descriptive labels were assigned to each cluster. Labels reflected the essential meaning and function of grouped concepts and were refined to be meaningful and understandable to the target audience (curriculum developers, educators, regulators). The finalized concept map organized the five themes into an integrated structure that guided results presentation and organization, discussion of implications for curriculum development and credentialing, and identification of future research needs and priorities.

## *Organization of 27 Codes into Five Thematic Clusters*

Through systematic concept mapping, the 27 individual codes were organized into five interconnected clusters representing key considerations for APRT educational preparation. Each cluster addresses a distinct dimension of APRT preparation while maintaining interconnectedness with the other clusters. Table 2 shows how the 27 codes were organized into the five final thematic clusters.

Table 2: Organization of 27 Codes into Five Thematic Clusters

| Thematic Cluster | Codes Included | Number of Codes |
| --- | --- | --- |
| Cluster 1: Content of Educational Preparation | Code 3: Leadership & research as key components; Code 6: Content of education; Code 7: Pillars of APRT (clinical, research, leadership, education); Code 17: Deepening the knowledge base beyond radiation therapist training; Code 18: Medical foundations for APRTs; Code 19: Specialized oncology knowledge | 6 |
| Cluster 2: Preparation Outcomes Aligned with Intended Scope of Practice | Code 1: Self-development, autonomy, self-actualization; Code 2: Role definition, distinction, development; Code 8: Autonomous critical thinking; Code 20: Strengthening clinical reasoning and decision-making skills; Code 21: Improving confidence in decision-making and managing uncertainty; Code 26: Promoting reflective practice and ongoing growth | 6 |
| Cluster 3: Diverse Learning Processes Integrating Multiple Pedagogical Approaches | Code 5: Systematic methods of assessment beyond competency; Code 11: Demonstration of learning; Code 13: Need to evidence AP through experiential learning & assessment; Code 22: Experiential learning: structured immersion in the clinical environment; Code 23: Structured clinical immersion; Code 24: Leading evidence-based care through research and quality improvement; Code 25: Communication across disciplines through collaborative leadership | 7 |
| Cluster 4: Key Requirements for Preparation | Code 4: Master's level education as the standard; Code 9: Level of education; Code 10: Work experience beyond entry-to-practice; Code 12: Common standard | 4 |
| Cluster 5: Contextual Factors Influencing Sustainability and Transportability | Code 14: Ability to serve the system; Code 15: Future-proofing: adaptability to changing practice; Code 16: Transportability: general vs specialization; Code 27: Adaptability and accessibility of APRT education program (including expert mentorship) | 4 |

### Cluster 1: Content of Educational Preparation

**Purpose:** Identifies the essential knowledge, skills, and competency domains that should be integrated into APRT educational programs.

This cluster captures the content and knowledge domains that participants identified as essential for APRT preparation. Consensus across all interest-holder groups emphasized a four-pillar approach integrating clinical practice expertise, research competency, leadership capability, and educational skill. Within the clinical pillar, participants stressed both breadth (understanding the full patient care pathway) and depth (specialized knowledge in the APRT's area of practice). Research competency was viewed as essential not merely as an academic exercise, but as a means to build evidence-based practice capability and contribute to service improvement. Leadership was distinct from management and encompassed being an independent thinker, effective team member, and change champion.

**Constituent Codes (n=6):** Code 3 (Leadership & research as key components); Code 6 (Content of education); Code 7 (Pillars of APRT (clinical, research, leadership, education)); Code 17 (Deepening the knowledge base beyond radiation therapist training); Code 18 (Medical foundations for APRTs); Code 19 (Specialized oncology knowledge)

### Cluster 2: Preparation Outcomes Aligned with Intended Scope of Practice

**Purpose:** Specifies the essential capabilities and scope of autonomous practice that APRT preparation should develop.

This cluster captures the outcomes APRTs should be able to achieve after preparation. Central to all discussions was the development of clinical decision-making competency that enables APRTs to integrate multiple information sources (clinical presentation, evidence, patient factors) to guide patient management. Participants emphasized that this went beyond technical competency to include autonomous critical thinking and the confidence to operate independently while maintaining appropriate accountability. The capability to manage uncertainty and complexity, combined with reflective practice, enables APRTs to adapt and grow throughout their career. Role clarity (understanding what distinguishes APRT from entry-level RT and the boundaries of autonomous practice) emerged as essential for sustainability and appropriate integration into healthcare teams.

**Constituent Codes (n=6):** Code 1 (Self-development, autonomy, self-actualization); Code 2 (Role definition, distinction, development); Code 8 (Autonomous critical thinking); Code 20 (Strengthening clinical reasoning and decision-making skills); Code 21 (Improving confidence in decision-making and managing uncertainty); Code 26 (Promoting reflective practice and ongoing growth)

### Cluster 3: Diverse Learning Processes Integrating Multiple Pedagogical Approaches

**Purpose:** Describes the varied methods, experiences, and mechanisms through which APRTs develop the knowledge and capabilities.

This cluster addresses the pedagogical approaches, learning activities, and assessment methods through which APRT competencies are developed and demonstrated. Participants consistently emphasized that no single learning method is sufficient. Rather, effective APRT preparation requires integration of didactic/formal learning (knowledge acquisition through classroom instruction), experiential/clinical learning (learning through direct patient care and practical immersion), project-based learning (leading quality improvement and research initiatives), and collaborative learning (working interdisciplinarily to develop communication and systems integration skills). Assessment approaches should go beyond assessing discrete competencies to evaluate capability (the integrated ability to apply knowledge, skills, and judgment in complex, real-world situations). This includes portfolio-based assessment, direct observation, case-based discussions, and multi-source feedback.

**Constituent Codes (n=7):** Code 5 (Systematic methods of assessment beyond competency); Code 11 (Demonstration of learning); Code 13 (Need to evidence AP through experiential learning & assessment); Code 22 (Experiential learning: structured immersion in the clinical environment); Code 23 (Structured clinical immersion); Code 24 (Leading evidence-based care through research and quality improvement); Code 25 (Communication across disciplines through collaborative leadership)

### Cluster 4: Key Requirements for Preparation

**Purpose:** Identifies the foundational qualifications, experience prerequisites, and formal requirements that define APRT preparation pathways.

This cluster identifies the formal structure and prerequisites associated with APRT preparation. There was strong consensus across all interest-holder groups that master's-level (graduate) education should be a standard requirement for APRT practice. Rationales included building foundational critical thinking and clinical decision-making skills, developing research literacy and evidence-based practice capability, establishing an externally recognized standardized credential, and socializing learners to self-directed learning. Equally important was extensive clinical experience in the intended practice area, with many participants suggesting that direct clinical apprenticeship under expert supervision may be as important as, or more important than, formal academic credentials. However, participants acknowledged practical barriers to mandating master's education, including limited volume of APRT positions in smaller jurisdictions, access and feasibility constraints, and variability in available programs and accreditation mechanisms. While standardization of core requirements is important for profession credibility and patient safety, implementation must account for contextual variability.

**Constituent Codes (n=4):** Code 4 (Master's level education as the standard); Code 9 (Level of education); Code 10 (Work experience beyond entry-to-practice); Code 12 (Common standard)

### Cluster 5: Contextual Factors Influencing Sustainability and Transportability

**Purpose:** Captures system-level, organizational, and regulatory factors that influence APRT role design, implementation, and sustainability.

This cluster addresses the broader context, recognizing that APRT preparation does not occur in isolation but within complex healthcare systems shaped by regulatory frameworks, funding mechanisms, institutional priorities, and workforce planning imperatives. Key contextual considerations include sustainability (APRT roles must be designed around autonomous practice and decision-making rather than around specific people or technologies), future-proofing (given rapid technological change, APRT preparation should emphasize adaptable, principle-based knowledge and critical thinking rather than training for specific current tools), transportability (tension between standardized APRT preparation enabling credential recognition and the practical reality that APRT scope must adapt to local context), system-level support (APRT sustainability requires supportive organizational infrastructure including mentorship, clear career pathways, and responsive credentialing frameworks), and career progression (APRT roles must offer meaningful career opportunities for experienced radiation therapists).

**Constituent Codes (n=4):** Code 14 (Ability to serve the system); Code 15 (Future-proofing: adaptability to changing practice); Code 16 (Transportability: general vs specialization); Code 27 (Adaptability and accessibility of APRT education program (including expert mentorship))

## Limitations of Analytical Approach

Research team-based clustering was conducted by the research team rather than with participant groups, potentially limiting interest-holder input into thematic organization. Some codes address multiple dimensions; placement in one cluster was based on primary emphasis but acknowledges secondary relevance elsewhere. Clustering reflects perspectives of 33 participants from 10 primarily English-speaking jurisdictions and may not fully represent low- and middle-income country contexts. The five-cluster model represents one point in time; future research may reveal different organizational patterns or additional clusters.

## Member Checking and Participant Feedback

Given the challenges of coordinating feedback from 33 internationally distributed participants, preliminary findings were discussed within the study team to solicit feedback on credibility and relevance. The detailed theme verification conducted by a team member (MD) not involved in initial coding served as an internal validity check. The five themes identified were systematically compared to published frameworks and literature on APRT preparation to assess external validity.
